# Shear Stress Accumulation Enhances von Willebrand Factor-Induced Platelet P-Selectin Translocation in a PI3K/Akt Pathway-Dependent Manner

**DOI:** 10.3389/fcell.2021.642108

**Published:** 2021-06-01

**Authors:** Jinhua Fang, Xiaoxi Sun, Silu Liu, Pu Yang, Jiangguo Lin, Jingjing Feng, Miguel A. Cruz, Jing-fei Dong, Ying Fang, Jianhua Wu

**Affiliations:** ^1^Institute of Biomechanics/School of Biology and Biological Engineering, South China University of Technology, Guangzhou, China; ^2^Research Department of Medical Sciences, Guangdong Provincial People’s Hospital, Guangdong Academy of Medical Sciences, Guangzhou, China; ^3^Cardiovascular Research Section, Department of Medicine, Baylor College of Medicine/Center for Translational Research on Inflammatory Diseases (CTRID), Michael E. DeBakey Veterans Affairs Medical Center, Houston, TX, United States; ^4^Bloodworks Research Institute and Hematology Division, Department of Medicine, University of Washington, Seattle, WA, United States

**Keywords:** platelet, P-selectin, shear stress, PI3K/Akt pathway, VWF-A1 domain and its mutants

## Abstract

Platelet adhesion and activation through the interaction of von Willebrand factor (VWF) with platelet glycoprotein (GP) Ibα are the early key events in hemostasis and thrombosis especially under high blood shear stress. P-selectin translocation from α granule to the cell surface is a typical platelet function phenotype, which makes the platelet-induced inflammatory response of flowing leukocytes possible and can be induced by either chemical agonists (thrombin, ADP, etc.) or high blood shear stress, but regulations of VWF mutation and blood shear stress on VWF-induced P-selectin translocation remain unclear. With flow cytometry, parallel plate flow chamber, and immunofluorescence staining techniques, we examined the P-selectin translocation of platelets on immobilized wild-type (WT) VWF-A1 domain and its two mutants, the gain-of-function (GOF) mutant R1308L and the loss-of-function (LOF) mutant G1324S, respectively. The results showed that the VWF-A1-induced platelet P-selectin translocation was triggered, accelerated, and enhanced by fluid shear stress and could be correlated with shear stress accumulation (SSA, the product of fluid shear stress and mechanical stimulus time), and the PI3K/Akt axis was involved in the platelet P-selectin translocation. The force-triggered P-selectin translocation occurred quickly on partial platelet surface first and then extended gradually to the whole platelet surface as SSA increased. The P-selectin translocation process would be promoted by the GOF mutation (R1308L) but slowed down by the LOF mutation (G1324S). These findings demonstrated a force-enhanced regulation mechanism for the VWF-induced platelet P-selectin translocation through the PI3K/Akt pathway and provided a novel insight into the mechano-chemical regulation mechanism for the key events, such as platelet activation and functional phenotype change in hemostasis and thrombosis.

## Introduction

Platelet activation is essential for thrombosis and hemostasis (Semple et al., 2011). P-selectin translocation is a key event that occurs in platelet activation and contributes to subsequent platelet aggregation and neutrophil recruitment (Ludwig et al., 2007; Korporaal et al., 2019; Tseng et al., 2019). Circulating platelets generally are at rest and coexist peacefully with endothelial cells under physiological conditions but can tether to, roll on, and adhere to injured vessel sites through the interaction of platelet glycoprotein (GP) Ibα to von Willebrand factor (VWF) which is secreted from the damaged vascular endothelial cells and ligated to the exposed subendothelial collagen (van der Meijden and Heemskerk, 2019). Once the recruited platelets are activated through transmembrane VWF-GPIbα signaling, P-selectin in platelet alpha particles can be expressed quickly on the platelet surface through membrane fusion (Furie et al., 2001; Gao et al., 2008; Li et al., 2010; Amelirad et al., 2019).

It is believed that soluble chemical agonists, such as thrombin and ADP as well as PAR1-AP, can induce platelet activation and P-selectin translocation through G protein-coupled receptors signaling in a dose-dependent manner in the absence of shear stress (Ivelin et al., 2019; St John et al., 2019; Schwarz et al., 2020), while prolonging the stimulus time will cause a decrease in platelet P-selectin surface density due to both the internalization and cleavage of P-selectin from the cell surface (Au and Josefsson, 2017). Besides the agonists, collagen can induce platelet P-selectin translocation through collagen-GPVI signaling (Ollivier et al., 2014), and shear stress does so (Lu and Malinauskas, 2011; Yin et al., 2011; Ding et al., 2015; Chen et al., 2016). The platelet P-selectin translocation would increase with applied shear stress and exposure time using a centrifugal flow-through Couette device (Chen et al., 2015). The P-selectin-positive platelet fraction can increase from 1.8 to 3.1% by applying a fluid load (shear rate of 1,000/s for 5 min) to whole blood with a cone-and-plate viscosimeter (Hu et al., 2003) but reaches 18.8% if shear stress of 70 dyn/cm^2^ is applied to platelets for 10 min (Roka-Moiia et al., 2020). High shear stress of 150 dyn/cm^2^ induced 12.8% P-selectin translocation after 5 min with a rotational viscometer and was enhanced with the increase of exposure time (Konstantopoulos et al., 1998; Merten et al., 2000). The response time of P-selectin translocation is not only agonist dependent (Whiss et al., 1998) but also force dependent. Fluid load (shear stress rate of 11,560/s) on whole blood makes the response time shorter than 1 s (Rahman and Hlady, 2019, 2021).

It is demonstrated that inhibiting VWF-GPIbα interaction can reduce effectively the force-induced platelet P-selectin translocation (Goto et al., 2000; Rahman and Hlady, 2021), while VWF-GPIbα signaling through PI3K/Akt participated in platelet integrin activation and high shear stress-induced platelet P-selectin translocation (Spater et al., 2018; Chen et al., 2019; Kral-Pointner et al., 2019). It is believed that catch–slip bond transition governs the interaction of VWF with GPIbα and retains but shifts the shear stress threshold in the cases of type 2B or 2M VWF mutations that result in bleeding disorders (Coburn et al., 2011). Pathological mutations on VWF might affect the binding of VWF to GPIbα. The gain of function (GOF) mutant R1308L has a higher binding affinity to GPIbα in comparison with wild-type (WT) VWF, and the loss-of-function (LOF) mutant G1324S does inversely (Baronciani et al., 2005; Morales et al., 2006; Auton et al., 2009; Federici et al., 2009). However, the regulation of both mechanical force and VWF mutation on platelet P-selectin translocation is not fully understood so far.

We herein studied the P-selectin translocation of platelets which were firmly adhered to a substrate coated with VWF-A1 or its two mutants (G1324S and R1308L) under various fluid shear stresses using parallel plate flow chamber (PPFC) and immunofluorescence staining. The present results showed that platelet P-selectin translocation was triggered, accelerated, and enhanced by shear stress but correlated with shear stress accumulation (SSA) instead of shear stress and mechanical stimulus time. Platelet P-selectin translocation was enhanced through GOF mutant R1308L but reduced by LOF mutant G1324S. The molecules PI3K and Akt were involved in the VWF-A1-induced P-selectin translocation. This work might provide a novel insight into the mechano-chemical regulation mechanism for the key early events in hemostasis and thrombosis.

## Materials and Methods

### Reagents

Apyrase, phorbol 12-myristate 13-acetate (PMA), Akt1/2 kinase inhibitor, and wortmannin (PI3 kinase inhibitor) were gained from Sigma-Aldrich (Burlington, MO, United States). FITC-conjugated anti-P-selectin antibody was purchased from AbD Serotec (Kidlington, Oxford, United Kingdom). The rabbit anti-human P-selectin polyclonal antibody was from Sino Biological, Inc. (Beijing, China), and the Alexa Fluor 488-conjugated goat anti-rabbit IgG secondary antibody was obtained from Thermo Fisher Scientific^TM^ (Waltham, MA, United States). All the other reagents were of analytical grade or the best grade available.

### Preparation of Washed Platelets

Washed platelets were prepared as reported elsewhere (Da et al., 2014). Briefly, blood was collected from healthy volunteers who had taken no medicine during the previous 2 weeks into vacuum blood collection tubes containing sodium citrate, according to the standard procedure. The blood was centrifuged at 200 × *g* for 10 min at room temperature. The platelet-rich plasma collected was supplemented with 5 U/ml apyrase to block platelet aggregation and centrifuged again at 1,000 × *g* for 10 min. The supernatant was removed, and the pellet was resuspended at a final concentration of 3 × 10^7^/ml in phosphate-buffered saline (PBS) containing 5% platelet-poor plasma.

### Expression and Purification of Recombinant VWF-A1 and Mutants

Recombinant VWF-A1 (amino acids Q1238–P1458) polypeptide was expressed in *Escherichia coli* as a fusion protein containing the His-tag in the N-terminus and purified as previously described (Cruz et al., 2000). Briefly, WTA1 protein was collected by centrifugation at 3,000 × *g* for 20 min at 4°C, resuspended in 50 mM phosphate buffer, pH 7.4, containing 500 mM NaCl, and sonicated. The clear lysate was applied to 5 ml HisTrap^TM^ HP column (GE Healthcare, Marlborough, MA, United States). After being washed with 50 mM sodium phosphate, 500 mM NaCl, and 20 mM imidazole at pH 7.4, the bound protein was eluted with the same buffer containing 150 mM imidazole. Mutant (R1308L and G1324S) plasmids were constructed by site-directed mutagenesis with Quick-Change II XL Site-Directed Mutagenesis kit (Agilent, Santa Clara, CA, United States) using the WTA1 plasmid as a template. The recombinant mutants were prepared as described for the WTA1 protein. The purified proteins were verified by 12% sodium dodecyl sulfate polyacrylamide gel electrophoresis (SDS-PAGE) and Western blot (data shown in Supplementary Figure 1). To verify the function of the purified proteins, the proteins were incubated overnight at a concentration of 200 μg/ml on petri dishes and then blocked with 2% bovine serum albumin (BSA) at room temperature for 1 h after washing with PBS thrice. The washed platelets were perfused at a shear stress of 1 dyn/cm^2^ for 5 min, and the number of adhering platelets at the last 1 min was recorded with an inverted microscope (Axio Observer A1, Zeiss, Oberkochen, Germany).

### Flow Cytometry

To confirm the role of shear stress in platelet P-selectin translocation induced by VWF-A1, the washed platelets were incubated in five distinct solutions with (i) PBS which served as a blank control, (ii) 1 μM of PMA as a positive control, (iii) 200 μg/ml of WTA1, (iv) 200 μg/ml of the mutant R1308L, and (v) 200 μg/ml of the mutant G1324S for 10 min at room temperature and then centrifuged at 1,000 × *g* for 5 min to remove excessive agonists. The platelets were resuspended and fixed in 2% paraformaldehyde for 30 min. After washing with PBS, the fixed platelets were blocked with 2% BSA at room temperature for 1 h. The PBS, PMA, WTA1, R1308L, or G1324S-treated platelets were incubated with a FITC-conjugated anti-P-selectin antibody in dark condition for 1 h. After washing with PBS, the samples were analyzed using flow cytometry (BD Biosciences, Franklin Lakes, NJ, United States). For each condition, three replicative experiments were performed.

### Immunostaining of Platelet P-Selectin in the Flow Assay

With the parallel-plate flow chamber (length × width × height = 20 mm × 2.5 mm × 0.254 mm) experimental system (Supplementary Figure 2), the platelets were loaded by shear stress. Purified VWF-A1 (WTA1, R1308L, and G1324S) were diluted with PBS to 200 μg/ml, respectively, and 20 μl of the solutions was added into petri dishes (Corning, NY, United States), which were held by a hollowed silicon gasket, marked in the cover slide center, and incubated overnight at 4°C. After washing with PBS containing 2% BSA thrice, the functionalized substrate was incubated with the same solution for 1 h at room temperature to block non-specific cell adhesion. Platelets (3 × 10^7^/ml) were perfused into the flow chamber. The washed platelets were incubated in petri dishes coated with WTA1, R1308L, or G1324S for 10 min and adhered to the petri dishes. At the end of the incubation, the platelets were stimulated by perfusing PBS at various fluid shear stresses for a range of time (0, 1, 2, 4, and 8 min). Prior to incubation with a rabbit anti-human P-selectin polyclonal antibody at 5 μg/ml in PBS containing 2% BSA, the stimulated platelets were fixed in 2% paraformaldehyde for 30 min at room temperature and subjected to saturation with PBS containing 2% BSA. An Alexa Fluor 488-conjugated goat anti-rabbit IgG secondary antibody at 4 μg/ml was used as a secondary antibody. To explore the signal pathway involved in the platelet P-selectin translocation induced by VWF-A1 in flows, washed platelets were pretreated with 1 μM wortmannin (Wymann and Arcaro, 1994) or 10 μM Akt1/2 kinase inhibitor (Sandstrom et al., 2019) for 10 min or 1 h at 37°C, respectively, before the perfusion. Observation and acquisition of differential interference contrast and fluorescence microscopy images were made using a fluorescence microscope. To investigate the platelet P-selectin translocation induced by shear stress without VWF-A1, the platelets were allowed to incubate on an empty petri dish for 10 min, and shear stress of 0 and 10 dyn/cm^2^ were applied to the platelets with a PPFC for 8 min, respectively. After fixing with 2% paraformaldehyde, the platelets were incubated with the rabbit anti-P-selectin antibody and goat anti-rabbit conjugated Alexa flour 488 secondary antibody, and the level of P-selectin translocation on the platelet surface was observed with a microscope. All images were processed with ImageJ software to derive the parameters.

### Character Parameters for Scaling Platelet P-Selectin Translocation

To quantify P-selectin translocation on activated platelet, three parameters, such as *F*_*P*_ (the fraction of P-selectin-positive platelets to total immobilized platelets on substrates) and *A*_*P*_ (the mean of P-selectin coverage area per P-selectin-positive platelet) as well as FI_*P*_ (the mean normalized platelet P-selectin-related fluorescence intensity per P-selectin-positive platelet), were chosen herein. With the immunofluorescence experimental data, the characteristic parameters *F*_*P*_, *A*_*P*_, and FI_*P*_ were calculated by

(1) $$F_{\text{P}}=\frac{N_{\text{positive}}}{N},A_{\text{P}}=\frac{A_{1}+A_{2}+\cdots +A_{n}}{N_{\text{positive}}}\,\mathrm{and}{\text{FI}}_{\text{P}}=\frac{{\text{FI}}_{1}+{\text{FI}}_{2}+\cdots +{\text{FI}}_{n}}{N_{\text{positive}}}\,\mathrm{with}\,{\text{FI}}_{\text{j}}=\frac{{\text{FI}}^{\left(j\right)}-{\text{FI}}_{\text{B}}}{{\text{FI}}_{\text{B}}}$$

*A*_*P*_, FI_*j*,_ and FI^(*j)*^ were the P-selectin coverage area, the P-selectin-related fluorescence intensity, and its normalized one of the *j*-th immobile platelet, respectively. *N*_*positive*_ and *N* represented the P-selectin-positive and total numbers of immobile platelets, respectively, and FI_*B*_ was the fluorescence intensity of the substrate background far away from each P-selectin-positive platelet. The fluorescent intensities on platelets were detected by Alexa Fluor 488-labeled antibody and used to estimate the platelet P-selectin translocation level.

### Statistics

Data are expressed as means ± standard error of the mean (SEM) from at least three independent experiments. Statistical significance was assayed by one-way (or two-way) ANOVA for multiple comparisons with a Bonferroni *post hoc* test (^∗^*p* < 0.05, ^∗∗^*p* < 0.01, ^∗∗∗^*p* < 0.001, and NS means non-significant).

## Results

### Force Is Necessary for VWF-A1-Induced Platelet P-Selectin Translocation

**FIGURE 1:**
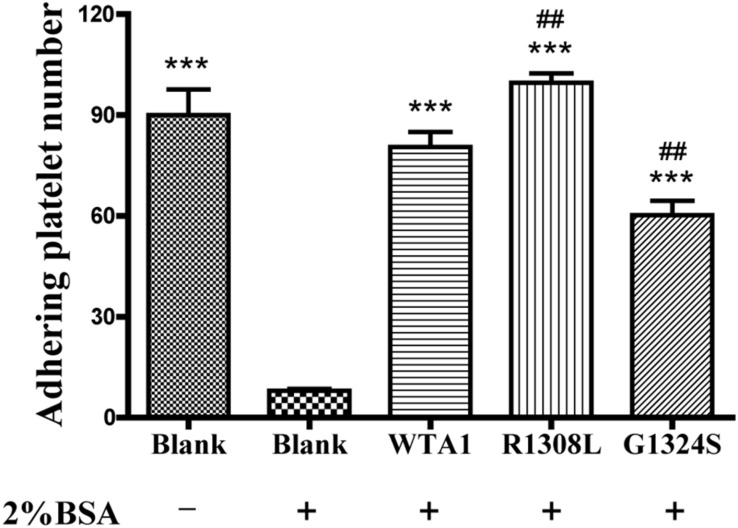
Specificity of platelet binding to substrates with or without VWF-A1 and its two mutants (R1308L and G1324S). The firmly adhering platelets on substrates with various treatments were numbered after perfusing phosphate-buffered saline over the substrates in a parallel plate flow chamber under a fluid shear stress of 1 dyn/cm^2^ for 5 min. The data represent the mean ± SEM from three independent experiments. Statistical significance was analyzed by one-way ANOVA for multiple comparisons with Bonferroni *post hoc* test; ***p < 0.001 compared with the blank group (treaded with 2% bovine serum albumin); ^##^*p* < 0.01 compared with the WTA1 group.

In studying the P-selectin translocation of platelets on VWF-coated substrates under flows, we first examined adhering platelets to substrates coated with or without 200 μg/ml VWF-A1 and/or its two mutants (R1308L and G1324S) at a fluid shear stress of 1 dyn/cm^2^ (“Materials and Methods”). The platelet adhesion was specific for VWF-A1 because of the high adhesion levels for platelet on VWF-A1 (WTA1, R1308L, and G1324S) in comparison with the low non-specific adhesion of platelet on substrates coated with the blank group treated with 2% BSA (Figure 1). The adhesion level of platelets on WTA1 was lower than that on R1308L mutant but higher than that on G1324S mutant, demonstrating both R1308L mutation-induced enhancement and G1324S mutation-induced reduction of platelet adhesions (Figure 1) as shown in previous works (Baronciani et al., 2005; Morales et al., 2006).

**FIGURE 2:**
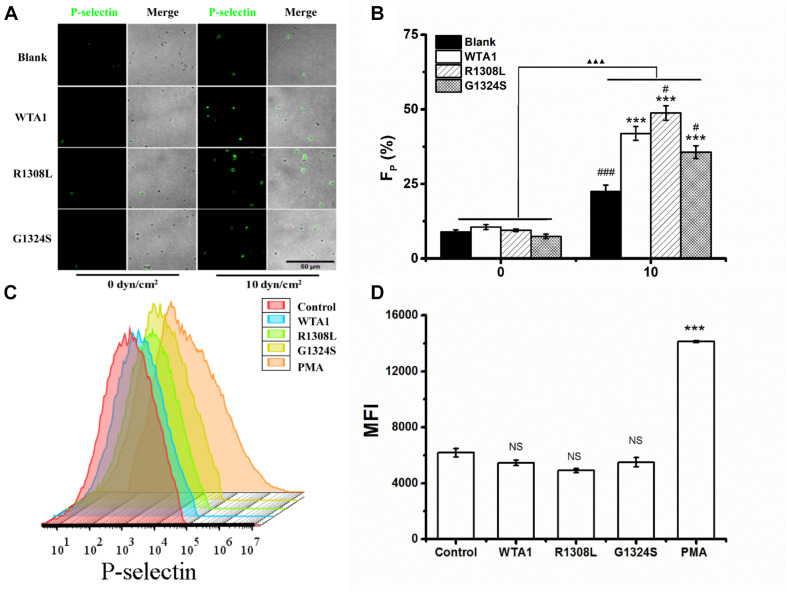
P-selectin translocation of platelets adhered to either immobilized- and suspended-A1 domain of von Willebrand factor and its two mutants (R1308L and G1324S) with or without mechanical stimuli for 8 min. **(A)** P-selectin immunolocalization (green) and **(B)** P-selectin-positive fraction (*F*_*P*_) of platelets on substrates coated with WTA1, R1308L, and G1324S without or with fluid shear stress stimulus of 10 dyn/cm^2^ for a stimulus time of 8 min. Merged images of differential interference contrast and green fluorescence are shown with bar = 50 μm. The data represent the mean ± SEM from three independent experiments. Statistical significance was analyzed by two-way ANOVA for multiple comparisons with Bonferroni *post hoc* test; ***p < 0.001 compared with the blank group, ^#^*p* < 0.05 and ^###^*p* < 0.001 compared with WTA1 group, and ^ΔΔΔ^*p* < 0.001 compared with 0 dyn/cm^2^ group. **(C)** Representative flow cytometry histograms of P-selectin translocation for platelets treated without (control) or with WTA1, R1308L, G1423S, and PMA. **(D)** Bar graph representing the mean fluorescence intensity of P-selectin-positive platelets (MFI) in various treatments from flow cytometry. All data are shown as mean ± SEM from three independent experiments and analyzed by one-way ANOVA for multiple comparisons. ***p < 0.001; NS, not significant compared with the control group.

To investigate the effect of external force on platelet P-selectin translocation, various fluid shear stresses were loaded to the firmly adhered platelets by perfusing PBS into the flow chamber (Materials and Methods). The rabbit anti-human P-selectin antibody was used to identify P-selectin-positive members of the immobilized platelets. We observed that P-selectin-positive platelets were rare for the platelets without mechanical preloads but became abundant for the platelets preloaded with fluid shear stresses of 10 dyn/cm^2^ for 8 min (Figures 2A,B). Force led to increases of the mean P-selectin-positive platelet fractions from 10.5 to 41.9, 48.8, and 35.6% for WTA1, R1308L, and G1324S, respectively (Figure 2B). PMA could induce platelet P-selectin translocation in suspension (Whiss et al., 1998) but could not with the VWF-A1 (WTA1, R1308L, and G1324S) because of no mechanical stimulus on the suspended platelets (Figures 2C,D). Besides this, we found that fluid shear stress induced only a small amount of P-selectin translocation in the absence of VWF-A1 (Figures 2A,B) as demonstrated in previous studies (Hu et al., 2003; Zhang et al., 2003). These data indicated that fluid shear stress and VWF synergistically regulated platelet P-selectin translocation.

### VWF-Mediated P-Selectin Translocation Was PI3K and Akt Dependent for Platelet Under Flows

**FIGURE 3:**
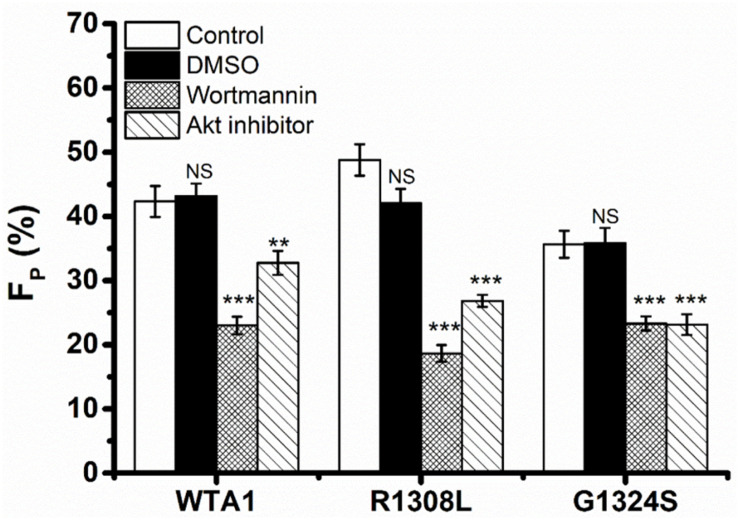
Effects of inhibition to PI3K and Akt on von Willebrand factor (VWF)-induced P-selectin translocation in flows. Fractions of P-selectin-positive inhibitor-treated or untreated platelets on VWF-A1 and its two mutants (R1308L and G1324S). The dimethyl sulfoxide group was from vehicle control experiments, but in the control and inhibitor groups, the P-selectin-positive platelets were treated without (blank column) inhibitor and with either wortmannin or Akt1/2 kinase inhibitor. Data of *F*_*P*_ (fraction of P-selectin-positive platelets) were from the independent inhibition experiments thrice under a fluid shear stress of 10 dyn/cm^2^ for a stimulus time of 8 min. All data are shown as the mean ± SEM and analyzed by one-way ANOVA for multiple comparisons. **p < 0.01, ***p < 0.001; NS, not significant compared with the respective control groups.

It was demonstrated that VWF-GPIbα signaling through PI3K and Akt was responsible for platelet integrin activation (Spater et al., 2018; Kral-Pointner et al., 2019). Thus, PI3K/Akt signaling might be involved in VWF-induced platelet P-selectin translocation because that P-selectin translocation was one of a series of platelet activation’s landmark events, such as platelet morphology changes, integrin activation, particle release (P-selectin translocation, etc.), and so on. We herein examined VWF-induced P-selectin translocation for platelets without pretreated (as control) and pretreated with PI3K inhibitor (wortmannin) or Akt inhibitor (Akt1/2 kinase inhibitor) under the fluid shear stress of 10 dyn/cm^2^ for 8 min (“Materials and Methods”). The data showed that there was no significant difference between the vehicle (DMSO) and controls (Figure 3) as it should be. The fraction of P-selectin-positive platelets decreased from 41.9 to 23.0 and 32.7% for treatments with wortmannin and Akt1/2 kinase inhibitors on substrates coated with WTA1, respectively (Figure 3), as did R1308L and G1324S. These results suggested VWF-GPIbα signaling through PI3K and Akt not only for platelet integrin activation but also for platelet P-selectin translocation (Figure 3). The reason might come from the fact that the P-selection translocation was a landmark event in platelet activation despite that there were some stimulators in platelet integrin activation and P-selectin translocation *via* different signal pathways (Kao et al., 2002; Li et al., 2010).

### Fluid Shear Stress Triggered, Accelerated, and Enhanced the Kinetics-Dependent VWF-A1-Induced P-Selectin Translocation of Platelets

Pathological mutations would affect the binding of VWF to platelet GPIbα, leading to flow-enhanced or flow-reduced adhesion of circulating platelets (Baronciani et al., 2005; Morales et al., 2006). For example, GOF mutant R1308L would strengthen the binding affinity of VWF-A1 domain to platelet GPIbα, and LOF mutant G1324S did the opposite. Fluid shear stress was demonstrated to be necessary for VWF-induced platelet P-selectin translocation, as shown in Figure 2. However, mechano-chemical regulation on P-selectin translocation of platelets on VWF under flows remained unclear.

**FIGURE 4:**
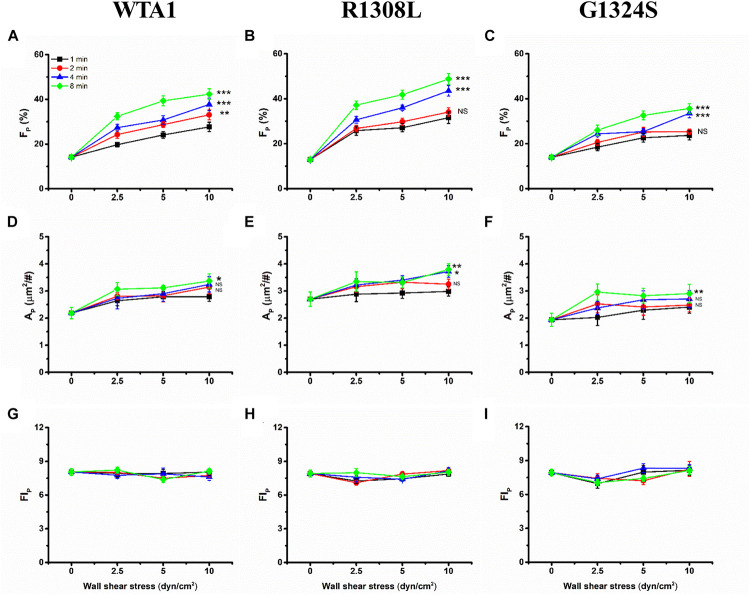
Variations of *F*_*P*_, *A*_*P*_, and FI_*P*_ of the immobilized platelets *versus* fluid shear stress. All the platelets on substrates coated with WTA1, R1308L, or G1324S were preloaded with fluid shear stress *τ*_*w*_ for mechanical stimulus times of 1, 2, 4, and 8 min. The mean P-selectin-positive platelet fraction *F*_*P*_ was plotted against fluid shear stress *τ*_*w*_ for platelet on WTA1, R1308L, and G1324S **(A–C)**, respectively, and so did either the mean of P-selectin coverage area *A*_*P*_ in **(D–F)** or the normalized platelet P-selectin fluorescence intensity FI_*P*_ in **(G–I)**. All data are shown as the mean ± SEM from at least three independent experiments and analyzed by two-way ANOVA for multiple comparisons; *p < 0.05, **p < 0.01, ***p < 0.001; NS, not significant compared with the 1-min group.

Through mechanical preloading treatments for platelets on the VWF-A1 (WTA1, R1308L, and G1324S)-coated substrates at fluid shear stresses of 0, 2.5, 5.0, and 10 dyn/cm^2^ for stimulus times of 0, 1, 2, 4, and 8 min (“Materials and Methods”), we herein examined the effects of mechanical stimulus and mutation on VWF-A1-mediated P-selectin translocation on the immobilized platelets. Three parameters, such as the P-selectin-positive platelet fraction *F*_*P*_ and the mean of P-selectin coverage area per positive platelets *A*_*P*_ as well as the mean normalized P-selectin-related fluorescence intensity per positive platelets FI_*P*_ (formula 1 in “Materials and Methods”), were exploited to weigh the platelet P-selectin translocation. The results were shown in the plots of *F*_*P*_, *A*_*P*_, and FI_*P*_ against fluid shear stress *τ*_*w*_ with various mechanical stimulus time *T* of 0, 1, 2, 4, and 8 min for platelet on the immobilized VWF-A1 (WTA1, R1308L, and G1324S) (Figure 4). For platelets on each of the immobilized VWF-A1, P-selectin translocation in the absence of shear stress stimulus (*τ*_*w*_ = 0) should be rare and weak due to the low P-selectin-positive fraction *F*_*P*_ and small P-selectin coverage area *A*_*P*_ (Figures 4A–F). Both *F*_*P*_ and *A*_*P*_ increased with fluid shear stress *τ*_*w*_ for each given stimulus time (0, 1, 2, 4, or 8 min), and increasing stimulus time *T* would cause an upswing of both *F*_*P*_ ∼ *τ*_*w*_ and *A*_*P*_ ∼ *τ*_*w*_ curves obviously, especially for *τ*_*w*_ < 5 dyn/cm^2^ (Figures 4A–F). It was suggested that fluid shear stress *τ*_*w*_ acted as an enhancer in platelet P-selectin translocation induced by VWF-A1, so did the mechanical stimulus time *T*. However, the FI_*P*_ ∼ *τ*_*w*_ curve fluctuated slightly with increasing of fluid shear stress *τ*_*w*_ for each given stimulus time, which meant that the P-selectin-related fluorescence intensity FI_*P*_ remained almost constant at different mechanical conditions (Figures 4G–I), suggesting a fast but local VWF-mediated P-selectin translocation on *A*_*P*_. These data suggested that mechanical stimulus served not only as a trigger but also as an accelerator or enhancer for VWF-induced translocation of platelet P-selectin, so did the mechanical stimulus time. LOF mutant (G1324S) reduced either *F*_*P*_ and *A*_*P*_ under diverse preloading, and GOF mutant (R1308L) did inversely, suggesting that VWF affinity to GPIbα regulated the force-dependent VWF-induced translocation of platelet P-selectin (Supplementary Figure 3).

### VWF-Induced Platelet P-Selectin Translocation Under Flows Could Be Correlated With Shear Stress Accumulation

**FIGURE 5:**
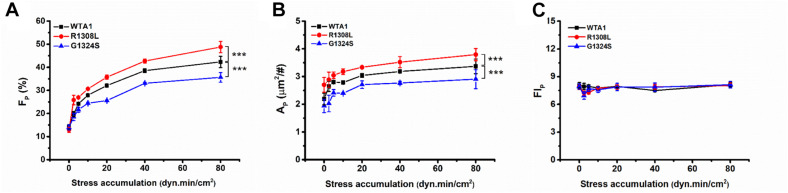
Regulation of shear stress accumulation on von Willebrand factor (VWF)-induced P-selectin translocation. The platelets firmly adhered to substrates coated with WTA1, R1308L, and G1324S, respectively. **(A)** The mean P-selectin-positive platelet fraction *F*_*P*_, **(B)** the mean of P-selectin coverage area *A*_*P*_, and **(C)** the normalized P-selectin-related fluorescence intensity FI_*P*_; all were well correlated with shear stress accumulation, whether VWF-A1 was mutated or not. All data are shown as mean ± SEM from at least three independent experiments and analyzed by two-way ANOVA for multiple comparisons; ***p < 0.001 compared with the WTA1 group.

Similar effects of fluid shear stress *τ*_*w*_ and mechanical stimulus time *T* on the VWF-A1-induced platelet P-selectin translocation were shown not only in *F*_*P*_ ∼ *τ*_*w*_ and *A*_*P*_ ∼ *τ*_*w*_ curves for each given *T* (Figure 4) but also in *F*_*P*_ ∼ T and *A*_*P*_ ∼ T curves for each given *τ*_*w*_ (Supplementary Figure 4). It indicated that *τ*_*w*_ and *T* might be similar in terms of efficacy but not independent in regulating the platelet P-selectin translocation despite that they were two independent determinants in the mechanical stimulus. These similar but synergistic effects of *τ*_*w*_ and *T* hinted that the shear stress accumulation, which denoted mechanical stimulus intensity and was the product of *τ*_*w*_ and T, might be a suitable biophysical parameter in scaling with the synergistic effects of *τ*_*w*_ and *T* on platelet P-selectin translocation (Sheriff et al., 2016). To examine this assumption, *F*_*P*_, *A*_*P*_, and FI_*P*_ were plotted against SSA for platelets on immobilized VWF-A1 (WTA1, R1308L, and G1324S) (Figure 5).

The plots of *F*_*P*_, *A*_*P*_, and FI_*P*_ against SSA (Figure 5) demonstrated that the VWF-A1-induced platelet P-selectin translocation could be correlated with SSA because all data for *F*_*P*_, *A*_*P*_, and FI_*P*_ at various fluid shear stresses and different stimulus times were clustered to the *F*_*P*_∼SSA, *A*_*P*_∼SSA, and FI_*P*_∼SSA curves, respectively (Figure 5 and Supplementary Tables 1–3). All of *F*_*P*_ in the same SSA values were very close and comparable (Supplementary Table 1), although the shear stresses and stimulation times were different. There was no coincidence that the fraction of P-selectin-positive platelets was similar at the same SSA because we could observe this phenomenon under all conditions with the same SSA value. *F*_*P*_ and *A*_*P*_ increased with SSA, while FI_*P*_ remained almost constant as SSA increased. The GOF mutation (R1308L) made both *F*_*P*_∼SSA and *A*_*P*_∼SSA curves upswing, and the LOF mutation (G1324S) did inversely, while these two different mutations had almost no effects on FI_*P*_ (Figure 5C and Supplementary Table 3). These results suggested a force-dependent VWF-induced platelet P-selection translocation process, in which the force-triggered P-selectin translocation occurred quickly but locally on the platelet surface with a small P-selectin coverage area *A*_*P*_ firstly and then extended gradually to the whole platelet surface with the increase of SSA. This process would be prompted by the GOF mutation (R1308L) but slowed down by the LOF mutation (G1324S). These results were consistent with previous work for SSA-prompted platelet activation using a modified prothrombinase assay (Sheriff et al., 2016).

## Discussion

As the first step for hemostasis, VWF recruits the circulating platelets to the vascular injured sites (Springer, 2014). The interaction of VWF to GPIbα initiates intracellular signal transduction to activate platelets and induce subsequent platelet P-selectin translocation in blood flows (Li et al., 2010; Amelirad et al., 2019). The P-selectin translocation could be induced also by both chemical agonists, such as thrombin, ADP, etc. (Ivelin et al., 2019; St John et al., 2019), and high shear stress (Ding et al., 2015; Chen et al., 2016) and contributed to platelet aggregation (Merten et al., 2000). The level of platelet P-selectin translocation is closely relevant with leukocyte inflammatory response, tumor cell hematogenous metastasis, hemostasis, thrombosis, and so on (Merten and Thiagarajan, 2004; Ludwig et al., 2007; Tseng et al., 2019; Schwarz et al., 2020), and VWF deficiency in quantity and quality can be caused by missense mutations and lead to bleeding and thrombotic von Willebrand diseases, such as microthrombosis found in patients with thrombotic thrombocytopenic purpura and bleeding found in patients with type 2B von Willebrand disease (Thiagarajan et al., 2013; Springer, 2014). Here, we studied VWF-A1-induced platelet P-selectin translocation under various shear stresses for different mechanical stimulus times using flow cytometry, parallel plate flow chamber, and immunofluorescence techniques. The present results demonstrated a mechano-chemical regulation mechanism on P-selectin translocation of platelets on substrates coated with VWF-A1 domain and its two mutants.

Previous studies demonstrated a force-dependent platelet P-selectin translocation through VWF-GPIbα signaling in flows (Rahman and Hlady, 2021), but for a low P-selectin translocation level of platelets in a high-shear-stress environment, it is said that high shear stress alone was likely to be insufficient in inducing platelet activation and aggregation (Zhang et al., 2003; Roka-Moiia et al., 2020). Our results also showed that the mechanical stimulus was necessary for VWF-A1 (including its two mutants)-induced platelet P-selectin translocation, but the level of platelet P-selectin translocation in the absence of VWF-A1 was significantly lower than that in the presence of VWF-A1 under all flow conditions (Figures 2A,B). The P-selectin translocation did not occur in either the suspended platelets (Figure 2D) or the immobilized platelets in the absence of shear stress (Figure 2B), suggesting that shear stress-mediated buildup of tension on VWF-A1/GPIbα complex might be required for platelet P-selectin translocation. The P-selectin-positive fraction and coverage area of platelets increased with either shear stress or stimulus time but could be correlated with shear stress accumulation instead of either fluid shear stress or mechanical stimulus time (Supplementary Table 1; Figures 4, 5). It is said that shear stress and stimulus time were not independent in inducing platelet P-selectin translocation and were coupled with each other by SSA, which coalesced out of the synergistic effects of shear stress and stimulus time on P-selectin translocation. So, serving as a crucial regulator, SSA promoted VWF-induced P-selectin translocation and activated the phenotype formation of platelets (Figures 5A,B). Platelet activation and P-selectin translocation shared with PI3K/Akt pathway in VWF-GPIbα signaling (Figure 3).

It was demonstrated that a mutation in the VWF-A1 domain might enhance or reduce platelet adhesion through changing VWF affinity to GPIbα, but less knowledge was relevant to the effects of VWF-A1 mutation on P-selectin translocation of the immobile platelets under shear stress. Similar to the previous work which found that the VWF cleaved by MMP-13 promoted the binding affinity to platelet and improved the platelet P-selectin translocation in comparison with intact VWF (Howes et al., 2020), our results showed that GOF mutant R1308L would cause an increment of about 10% in either P-selectin-positive platelet fraction or P-selectin coverage platelet area, while LOF mutant G1324S led to a reduction of 10% at least in either P-selectin-positive platelet fraction or P-selectin coverage platelet area in comparison with WTA1 for SSA larger than 5 dyn⋅min/cm^2^ (Figure 5), suggesting the VWF affinity (to GPIbα)-dependent platelet P-selectin translocation. These findings indicated that GOF mutant R1308L would facilitate platelet aggregation, hemostasis, and thrombosis through enhancing not only platelet adhesion but also P-selectin translocation which were demonstrated to be dominant in platelet aggregation and thrombosis (Konstantopoulos et al., 1998; Merten et al., 2000), and LOF mutant G1324S did the opposite. These data further argued that R1308L was a GOF mutation instead of a LOF one, which was consistent with previous works for platelet adhesion (Baronciani et al., 2005). In contrast, one recent study classified R1308L to a LOF mutation based on the fact that the pause time of platelets on R1308L substrate under the shear rate of 1,500s^–1^ was less than that on WTA1 (Tischer et al., 2014). Perhaps the upregulated associated rate could counteract fully the effects of the upregulated dissociation rate on VWF affinity to GPIbα. This phenomenon also occurred in the GOF mutation R1450E. The ability of R1450E mutant to recruit platelets was significantly higher than that of WTA1 (Morales et al., 2006), but this mutation would make the mean stop time short (Coburn et al., 2011).

In conclusion, our data suggested that VWF-induced platelet P-selectin translocation would be triggered, accelerated, and enhanced by fluid shear stress but correlated with shear stress accumulation. This SSA-dependent P-selectin translocation of platelets would be enhanced by GOF mutant R1308L but reduced through LOF mutant G1324S. The present work provided a novel insight into the mechano-chemical regulation on adhesive molecule-mediated platelet P-selectin translocation and its relevant biological processes, such as tumor cell hematogenous metastasis, hemostasis, and inflammatory responses under flows.

## Data Availability Statement

The original contributions presented in the study are included in the article/Supplementary Material, further inquiries can be directed to the corresponding authors.

## Ethics Statement

The studies involving human participants were reviewed and approved by the Guangzhou First People’s Hospital. The patients/participants provided their written informed consent to participate in this study.

## Author Contributions

JW and YF designed this research. JFa supervised this study overall. XS, SL, PY, JFe, JL, MAC, and J-FD partly performed research and analyzed data. JFa, YF, and JW wrote this manuscript. All authors contributed to the article and approved the submitted version.

## Conflict of Interest

The authors declare that the research was conducted in the absence of any commercial or financial relationships that could be construed as a potential conflict of interest.
