# Supplementary material for: Shear Stress Accumulation Enhances von Willebrand Factor-Induced Platelet P-Selectin Translocation in a PI3K/Akt Pathway-Dependent Manner
Source: Front Cell Dev Biol. 2021 Jun 1;9:642108. doi: 10.3389/fcell.2021.642108 (PMC8204100; doi:10.3389/fcell.2021.642108)
Supplement: Supplementary file 1 [file Data_Sheet_1.docx]

Supplementary Material

# Supplementary Figures and Tables

## Supplementary Figures

##
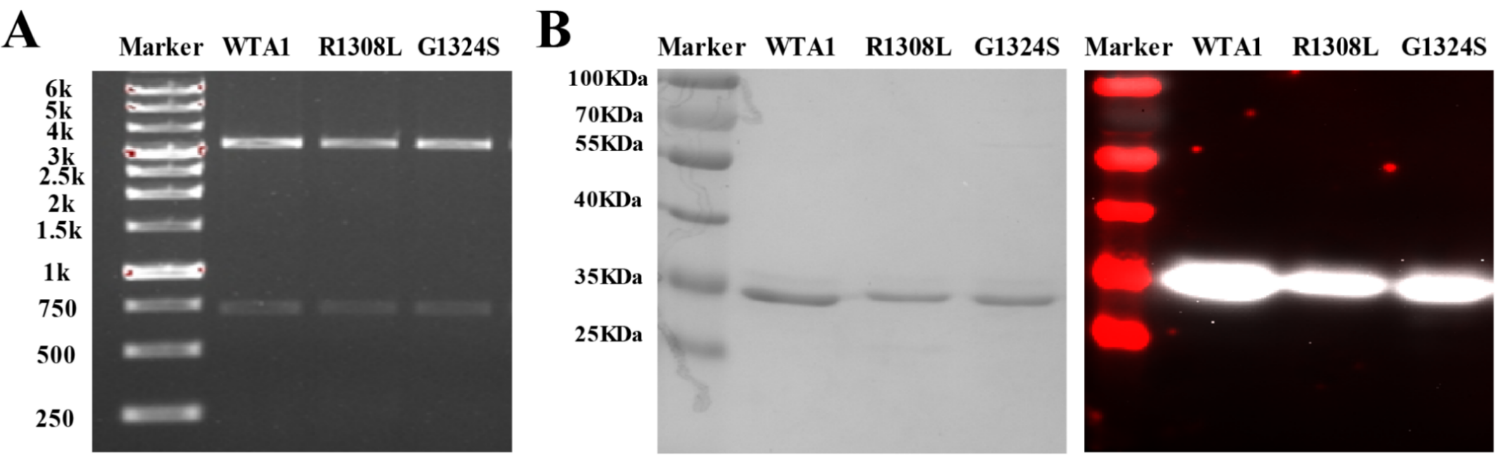


**Supplementary Figure 1. Preparation of recombinant VWF-A1 and its mutants.** (A) WTA1, R1308L, or G1324S plasmids were double digested by restriction enzymes BamHI and HindIII, and verified in agarose gel electrophoresis. (B) Purified WTA1, R1308L, or G1324S were analyzed by SDS-PAGE and Western blotting. Rabbit anti-6×His antibody was used to identify the VWF-A1 with 6 His-tags on the membrane. Goat anti-rabbit IgG conjugated HRP antibody was used as a secondary antibody to recognize rabbit anti-6×His antibody, which can catalyze the chemiluminescence of ECL substrate. The 26616 marker was a reference for protein molecular weight.


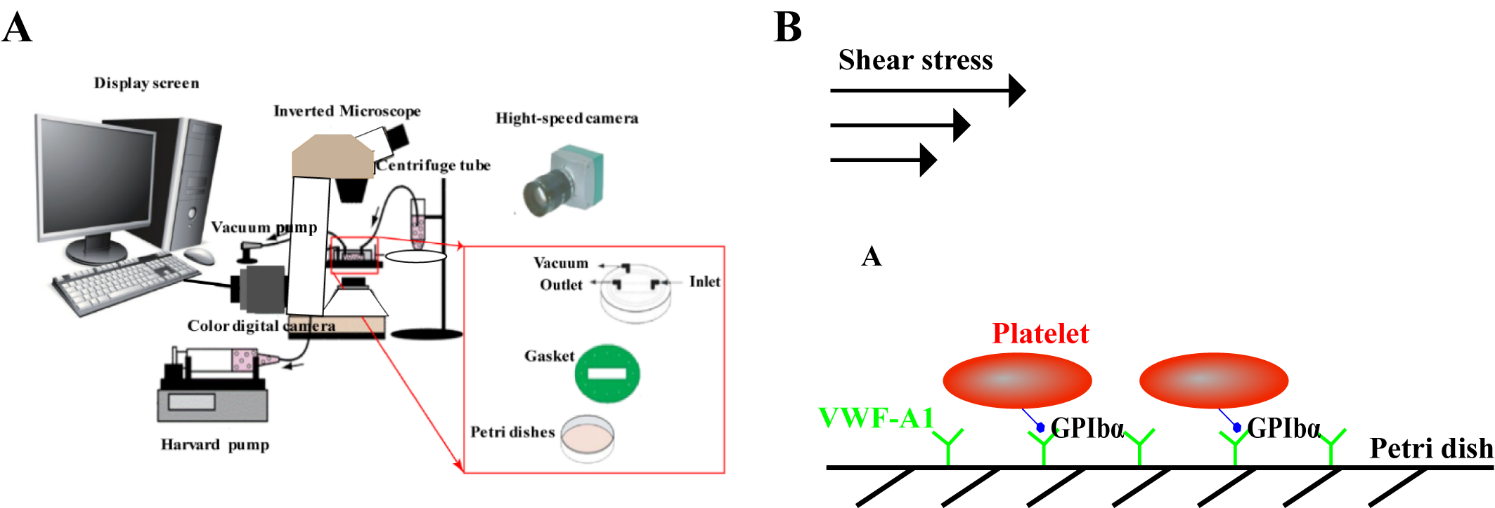


**Supplementary Figure 2. The system of the parallel plate flow chamber.** (A) The parallel plate flow chamber system consists of five parts: inverted microscope, fluorescence camera, perfusion device (Harvard pump), image analysis software, and flow chamber. (B) Schematic diagram of the functionalization of the flow chamber.


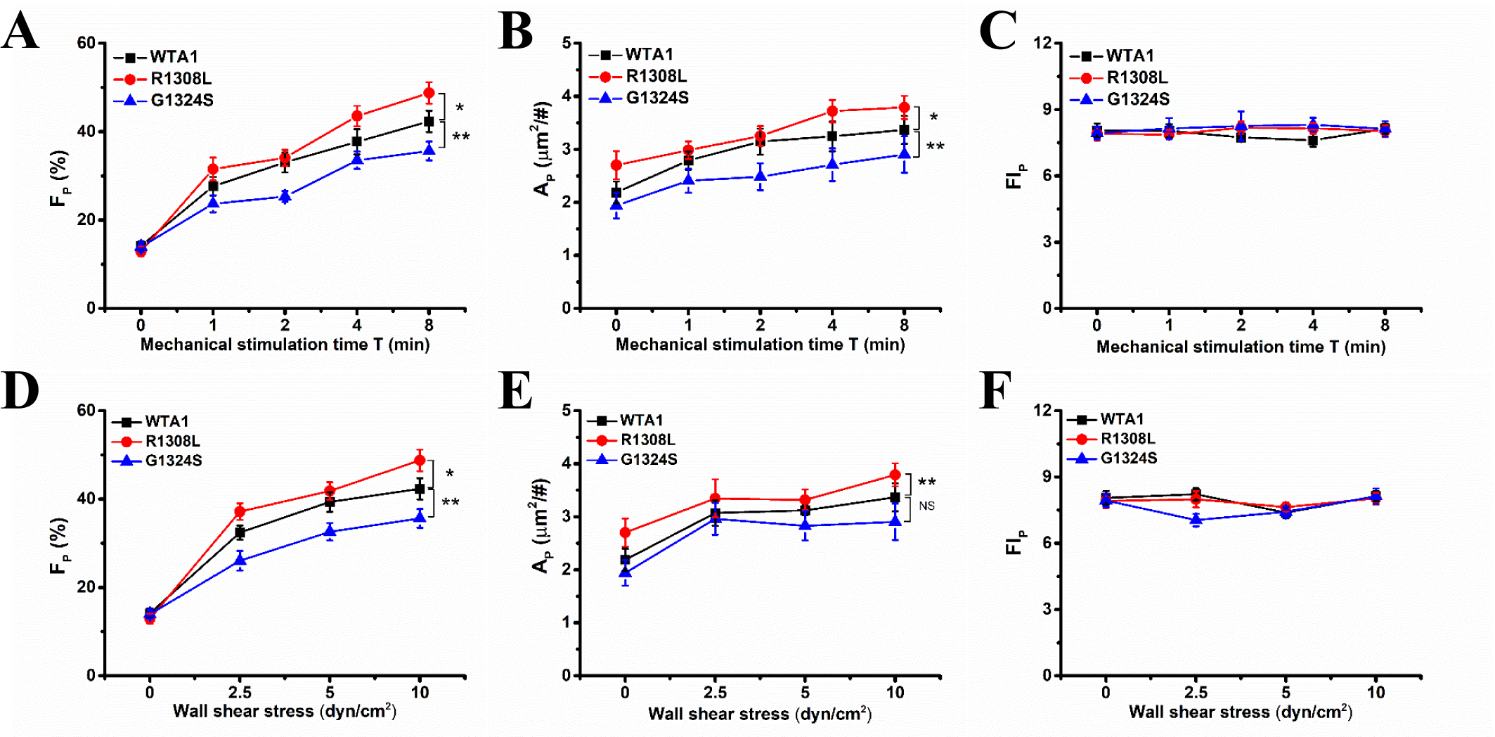


**Supplementary Figure 3. Variation of VWF-induced platelet P-selectin translocation versus either fluid shear stress or mechanical stimulus time.** The platelets firmly adhered to substrates coated with WTA1, R1308L, and G1324S respectively. **(A-C)** Plots of F_P_ (the P-selectin positive platelet fraction) and A_P_ (the mean of P-selectin coverage area per positive platelet), as well as FI_P_ (the normalized platelet P-selectin fluorescence intensity) against T (the mechanical stimulus time) for platelets on WTA1, R1308L, and G1324S at fluid shear stress τ_w_ of 10 dyn/cm^2^. **(D-F)** Variation of F_P_, A_P_, and FI_P_ versus τ_w_ for platelets on WTA1, R1308L, and G1324S using mechanical stimulus time of 8min. All data were shown as the mean ± SEM from at least three independent experiments and analyzed by two-way ANOVA for multiple comparisons, * for p < 0.05 and ** for p<0.01 compared with the WTA1 group.


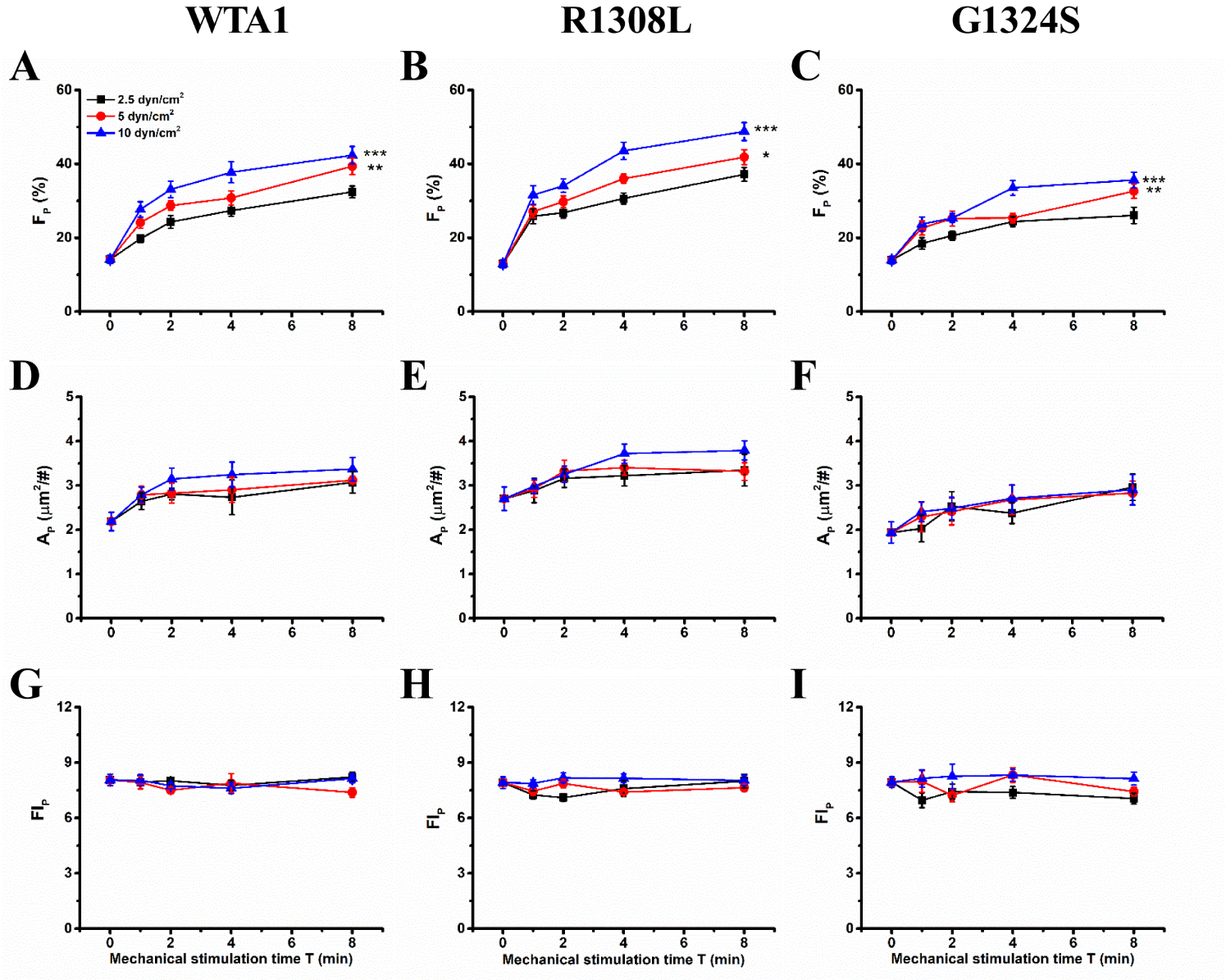


**Supplementary Figure 4. Variations of F_P_, A_P,_ and FI_P_ of the immobilized platelets versus mechanical stimulus time under various fluid shear stress τ_w_.** All the platelets on substrates coated with WTA1, R1308L, or G1324S were preloaded with various fluid shear stress τ_w_ for different mechanical stimulus times. The mean P-selectin-positive platelet fraction F_P_ was plotted against mechanical stimulus time for platelets on WTA1, R1308L, and G1324S and shown in **(A-C)** respectively, so did either the mean of P-selectin coverage area A_P_ in **(D-F)** or the normalized platelet P-selectin fluorescence intensity FI_P_ in **(G-I)**. All data were shown as the mean ± SEM from at least three independent experiments and analyzed by two-way ANOVA for multiple comparisons; * for p < 0.05, ** for p<0.01, and *** for p<0.001 compared with the 2.5 dyn/cm^2^ group.

## Supplementary Tables

Table1. The fraction of P-selectin positive platelets induced by different VWF-A1 molecules (WTA1, R1308L, and G1324S) at different force conditions.

| **Shear stress**  **(dyn/cm^2^)** | **Time**  **(min)** | **SSA**  **(dyn.min/cm^2^)** | **F_P_ (%)** | | |
| --- | --- | --- | --- | --- | --- |
|  |  |  | **WTA1** | **R1308L** | **G1324S** |
| **2.5** | **2** | **5** | **24.28** | **26.78** | **20.58** |
| **5** | **1** | **5** | **24.08** | **27.12** | **22.66** |
| **2.5** | **4** | **10** | **27.36** | **30.64** | **24.39** |
| **5** | **2** | **10** | **28.71** | **29.75** | **25.22** |
| **10** | **1** | **10** | **27.66** | **31.56** | **23.69** |
| **2.5** | **8** | **20** | **32.41** | **37.17** | **26.06** |
| **5** | **4** | **20** | **30.79** | **36.00** | **25.37** |
| **10** | **2** | **20** | **33.08** | **34.10** | **25.33** |
| **5** | **8** | **40** | **39.35** | **41.83** | **32.61** |
| **10** | **4** | **40** | **37.72** | **43.54** | **33.52** |

SSA, shear stress accumulation, the product of shear stress and mechanical stimulus time; WTA1, wild-type of VWF-A1; R1308L, gain-of-function mutant of VWF-A1; G1324S, loss-of-function mutant of VWF-A1; F_P_, the fraction of P-selectin positive platelets, each color represents an SSA value.

Table2. The mean of P-selectin coverage area per positive platelet induced by different VWF-A1 molecules (WTA1, R1308L, and G1324S) at different force conditions.

| **Shear stress**  **(dyn/cm^2^)** | **Time**  **(min)** | **SSA**  **(dyn.min/cm^2^)** | **A_P_ (μm^2^/#)** | | |
| --- | --- | --- | --- | --- | --- |
|  |  |  | **WTA1** | **R1308L** | **G1324S** |
| **2.5** | **2** | **5** | **2.81** | **3.16** | **2.53** |
| **5** | **1** | **5** | **2.79** | **2.93** | **2.29** |
| **2.5** | **4** | **10** | **2.74** | **3.22** | **2.38** |
| **5** | **2** | **10** | **2.82** | **3.32** | **2.41** |
| **10** | **1** | **10** | **2.79** | **2.99** | **2.41** |
| **2.5** | **8** | **20** | **3.07** | **3.35** | **2.96** |
| **5** | **4** | **20** | **2.90** | **3.40** | **2.68** |
| **10** | **2** | **20** | **3.15** | **3.25** | **2.48** |
| **5** | **8** | **40** | **3.12** | **3.32** | **2.83** |
| **10** | **4** | **40** | **3.25** | **3.72** | **2.71** |

SSA, shear stress accumulation, the product of shear stress and mechanical stimulus time; WTA1, wild-type of VWF-A1; R1308L, gain-of-function mutant of VWF-A1; G1324S, loss-of-function mutant of VWF-A1; A_P_, the mean of P-selectin coverage area per positive platelet, each color represents an SSA value.

Table3. The mean normalized platelet P-selectin-related fluorescence intensity induced by different VWF-A1 molecules (WTA1, R1308L, and G1324S) at different force conditions.

| **Shear stress**  **(dyn/cm^2^)** | **Time**  **(min)** | **SSA**  **(dyn.min/cm^2^)** | **FI_P_** | | |
| --- | --- | --- | --- | --- | --- |
|  |  |  | **WTA1** | **R1308L** | **G1324S** |
| **2.5** | **2** | **5** | **8.01** | **7.10** | **7.42** |
| **5** | **1** | **5** | **7.92** | **7.45** | **7.98** |
| **2.5** | **4** | **10** | **7.76** | **7.59** | **7.38** |
| **5** | **2** | **10** | **7.50** | **7.87** | **7.22** |
| **10** | **1** | **10** | **8.04** | **7.87** | **8.15** |
| **2.5** | **8** | **20** | **8.22** | **7.99** | **7.05** |
| **5** | **4** | **20** | **7.88** | **7.40** | **8.33** |
| **10** | **2** | **20** | **7.74** | **8.18** | **8.26** |
| **5** | **8** | **40** | **7.38** | **7.64** | **7.42** |
| **10** | **4** | **40** | **7.61** | **8.15** | **8.32** |

SSA, shear stress accumulation, the product of shear stress and mechanical stimulus time; WTA1, wild-type of VWF-A1; R1308L, gain-of-function mutant of VWF-A1; G1324S, loss-of-function mutant of VWF-A1; FI_P_, the mean normalized platelet P-selectin-related fluorescence intensity, each color represents an SSA value.
